# Quality of inpatient test-and-treat malaria case-management in public and private hospitals in Kano State, Nigeria

**DOI:** 10.1186/s12936-025-05742-7

**Published:** 2026-02-07

**Authors:** Dawit Getachew, Nnenna Ogbulafor, Emmanuel Shekarau, Babangida Musa, Abdullahi Yusuf, Safiyanu Haruna, Oladipo O. Oladosu, Olusola Oresanya, Kolawole Maxwell, Dejan Zurovac

**Affiliations:** 1 Malaria Consortium, Abuja, Nigeria; 2 National Malaria Elimination Programme, Abuja, Nigeria; 3 State Malaria Elimination Programme, Kano, Nigeria; 4 Bowen University Iwo, Iwo, Osun Nigeria; 5 Independent Consultant, Zagreb, Croatia

**Keywords:** Malaria, Quality, Compliance, Guidelines, Inpatients, Nigeria

## Abstract

**Background:**

Compliance with evidence-based treatment guidelines, supported by quality-assured parasitological diagnosis, is the mainstay of malaria case-management in Nigeria. However, despite increased attention, the quality of inpatient paediatric and adult, test-and-treat malaria case-management, and routine accuracy of malaria microscopy, has rarely been examined in public and private hospitals.

**Methods:**

A cross-sectional assessment was undertaken at 18 public and private hospitals in September 2024 in Kano State, Nigeria. Data collection included hospital assessments, interviews with inpatient health workers, review of all paediatric and medical ward admission files for August 2024, and re-checking of routine malaria slides archived during the 3-month post-assessment period. Descriptive analyses included 18 hospitals, 72 health workers, 2,814 suspected malaria admissions, and 211 malaria slides.

**Results:**

Nearly all hospitals (94.4%) provided parasitological diagnostic services (microscopy or RDT) and stocked recommended antimalarials (injectable artesunate and ACT). Most health workers had received training on severe malaria (73.6%), but only 16.7% received supportive supervision. The composite test-and-treat performance was 39.3%, higher for children than adults (45.7% vs 26.5%) and in public compared to private hospitals (39.8% vs 30.8%). Among suspected malaria patients, 73.7% were tested on admissions and 90.2% of those with severe malaria were treated with artesunate. Children, compared to adults, were more commonly tested (79.8% vs 61.7%) and treated with artesunate (93.5% vs 80.1%). Patients in private hospitals, compared to public, were more often tested (84.3% vs 73.0%) but less frequently treated with artesunate (73.1% vs 91.2%). Only 30.0% of artesunate-treated patients were prescribed ACT—more commonly among adults than children (48.3% vs 23.0%) and in private than in public hospitals (89.2% vs 26.9%). ACT use for admitted non-severe cases was rare (2.4%), whereas non-compliance with test negative results was high (75.8%). The sensitivity, specificity, positive and negative predictive values of routine microscopy compared to expert readings were 93.2%, 42.5%, 29.9% and 95.9%, respectively.

**Conclusions:**

Inpatient compliance with malaria test-and-treat guidelines varied between performance tasks, age groups, and hospital sectors. Clinicians can be confident in negative slides but should be cautious with positive results. Quality assurance of malaria diagnosis and continuous clinical and laboratory quality improvement interventions, with enhanced linkages, are needed.

## Background

Nigeria bears the highest malaria burden globally, accounting for 26% of all malaria cases and 31% of malaria deaths [1]. The disease burden on the health system is similarly high with nearly a third of admissions in Nigerian hospitals due to malaria [2]. Compliance with evidence-based treatment guidelines, supported by quality-assured parasitological diagnosis, has been the mainstay of malaria case-management since the adoption of targeted test-and-treat recommendations in 2010 [3, 4]. A paradigm shift in the management of severe malaria took place in 2012, when the national treatment policy was changed from quinine to parenteral artesunate—the therapeutic option recommended by the World Health Organization (WHO) and shown to reduce malaria mortality in multicentre trials, including those undertaken in Nigeria [5–7].

After decades of neglect of severe malaria [8], the launch of the new treatment policy provided an opportunity to strengthen hospital systems, parasitological diagnosis and broader aspects of inpatient quality of care for malaria in line with national guidelines [9–11]. However, despite the renewed interest in severe malaria, the life-saving importance of the quality of care [12], the demonstrated impact of compliance with malaria case-management guidelines [13, 14], and programmatic interventions deployed at various scales countrywide [15], little is routinely known in Nigeria about clinical practices for patients admitted with suspected malaria. The complexity of data collection reflecting quality of inpatient care processes spreading over multiple admission days contributes to this lack of insight. However, when special studies are undertaken sporadically, suboptimal quality has been observed. Specifically, non-compliance with guidelines, characterized by suboptimal testing rates of febrile patients on admission; presumptive treatments without testing or despite negative test results; lack of parasitological monitoring; use of non-recommended antimalarials; irrational use of parenteral antimalarials for non-severe cases and incomplete treatment for severe malaria are few of inpatient clinical deficiencies found in Nigeria [16–18], as well as in other African countries [19–25]. Moreover, despite receiving increased attention in recent years, reports on inpatient malaria case-management remain skewed towards paediatric care, often undertaken at single sites and tertiary settings, rarely addressing the adult population and focusing mainly on public sector hospitals.

Furthermore, the accuracy of parasitological results, in particular malaria microscopy in hospital settings, is the backbone of quality-assured malaria diagnosis [26], a critical component of the clinical process determining the credibility of targeted malaria test-and-treat policy, compliance of clinicians with malaria case-management guidelines, monitoring of treatment response, and patient outcomes for severe febrile illness [27, 28]. While microscopy has been used for decades across Africa and concerns about its quality have long been raised [29–31], only more recent studies—none of them from Nigeria— have reported higher accuracy of routine microscopy [32, 33].

In this manuscript, the findings from public and private hospitals in Kano State, Nigeria where the quality of inpatient malaria case-management—both paediatric and adult—as well as the accuracy of routine malaria microscopy, are reported.

## Methods

### Description of study area

Kano State is commercial and agricultural region located in the northwest geopolitical zone of Nigeria, occupying a surface area of 20,760 km^2^ and having a projected population of approximately 16 million in 2025. The climate is tropical wet and dry, typical of the West African savanna, with malaria transmission occurring throughout the year and seasonal peaks following rainy season, usually from May to October. In 2021, *Plasmodium falciparum* prevalence rate measured by malaria microscopy in children 6–59 months during high malaria season was estimated at 26% [34]. Malaria incidence in the State was estimated at 398 per 1000 population at risk and case burden contributed to 9% of Nigeria’s 68 million malaria cases in 2021 [35].

### Health systems context

Inpatient services in Kano State are provided by 39 secondary and two tertiary hospitals of which six are privately owned and located within the Kano metropolis. Most hospitals admit paediatric and non-pregnant adult patients through outpatient and hospital emergency units. Laboratories provide diagnostic support by doing malaria rapid diagnostic tests (RDT) and microscopy. Malaria microscopy is conducted by medical laboratory scientists while outpatient, emergency and inpatient services are provided and overseen by medical doctors and supported by nurses. The quality of inpatient malaria case-management at public hospitals is supported mainly by donor funded programmatic activities, including a) the procurement and distribution of RDTs, injectable artesunate and artemisinin-based combination therapy (ACT), which are issued at no cost to patients, b) series of in-service trainings on malaria case-management delivered over years to the front line health workers, c) provision of occasional malaria microscopy trainings for laboratory personnel and irregular external quality assurance activities for parasitological malaria diagnosis, d) delivery of malaria integrated supportive supervision using standardized checklist, and e) distribution of guidelines and job aids to health workers [15, 36, 37]. Private hospitals in contrast are for-profit health service entities that are not targeted with government programmatic support activities.

### Study populations

The assessment of the quality of inpatient test-and-treat malaria case-management and accuracy of malaria microscopy was cross-sectional, undertaken at 18 hospitals in Kano State, purposively selected to reflect diverse State’s representation, presence of paediatric and medical ward admission services, availability of malaria microscopy, inclusion of private sector hospitals, and assessment costs. The assessment populations included hospital departments of relevance for inpatient malaria case-management, health workers from paedatric and medical wards, and case files for patients admitted in paediatric and medical wards. Specifically, all hospitals were assessed, one clinician and one nurse from paediatric and medical wards who were on day-shift duty during the first assessment day were interviewed; and clinical data were extracted from admission files meeting criteria of suspected malaria during the one-month period prior to data collection, i.e. from 1st-31st August 2024. The period for data extraction coincided with the peak of malaria season in Kano State. Due to the very low numbers of archived routine malaria slides during the baseline assessment, blood slides intended for rechecking by expert microscopists were collected post-assessment, three months later and included slides archived between September and November 2024.

### Data collection and procedures

Data collection at each hospital was performed over five working days in September 2024 by the hospital resident team composed of a medical records officer and a nurse with inpatient experience. The hospital teams were supervised by external study clinicians, who also facilitated theoretical and practical three-day data collection training in the week prior to the field work. During hospital data extraction, records officers first undertook counts of all admissions to the paediatric and medical wards within the study period and thereafter retrieved all case files corresponding to the same patients. All retrieved files were screened to identify patients with suspected malaria, defined as documentation on admission of the history of fever, temperature ≥ 37.5 °C, diagnosis of malaria or prescription of antimalarial treatment. From the suspected malaria case files, data elements were extracted from structured and unstructured admission, continuation, laboratory, observation, treatment, and discharge forms. The main extracted data elements included age, sex, weight, dates of admission and discharge, assessments and laboratory tests performed, test results and diagnoses made, and treatments prescribed during the hospitalization and on discharge. The presence of clinical criteria for severe malaria on admission was established as documented in the emergency unit, outpatient department, or within 24 h of admission to the ward.

The second data collection method included interviews and knowledge assessments of randomly selected health workers on duty in the paediatric and medical wards. If, on the first day of data collection, only one doctor or nurse was covering both wards of interest, an additional health worker was selected from the next shift. The interviews and knowledge assessments, after providing written informed consent, lasted about 15–20 min and were arranged at convenient time for the interviewees. During the interviews retrospective exposure to malaria case-management support interventions was established while health workers´ knowledge about inpatient test-and-treat malaria standards and artesunate use was assessed using self-administered, multiple-choice questions with single select response options. Thirdly, physical assessments of the availability of antimalarial medicines and RDTs, displayed job aids, malaria microscopy services, and basic equipment were conducted on the first day of data collection across hospital departments including paediatric and medical wards, pharmacy, laboratory and emergency units.

Finally, in line with national protocols for external quality assurance of parasitological malaria diagnosis [9], ten routine low-positive and five negative malaria slides archived by hospital laboratories were randomly selected for off-site rechecking by WHO level 1 certified expert microscopists. Two expert microscopists blindly rechecked the slides, and a third independent expert microscopist served as a tie-breaker in the case of discordant results. The third reading was required for 3% of the slides. Expert readers were blinded to both the routine results and the results of the other expert microscopists. The microscopists examined a minimum of 100 high-power magnification fields before each slide was classified as negative.

### Indicators and analysis

Key indicators were aligned with standards for the management of suspected malaria admissions specified in the national malaria case-management and quality of care guidelines [10, 11], with main test-and-treat compliance domains reflecting quality of care, and accounting for relatively simple data collection methods such as case file data extraction, interviews and hospital assessments. Primary indicators were measured at patient level and reflected compliance with critical test-and-treat case-management standards in Nigeria specifying that patients admitted with suspected malaria should be parasitologically tested for malaria and depending on the severity criteria and test result should have either parenteral artesunate prescribed for test positive severe cases (i.e. confirmed severe malaria), ACT for non-severe positive patients (i.e. uncomplicated malaria) or no antimalarial for test-negative patients. Severity features among suspected malaria admissions were determined operationally as either documentation of any guideline-recommended criteria on admission, or—recognizing the limitations in documenting of clinical features—by health workers’ diagnosis of severe malaria made at admission. Further patient level indicators referring to the performance of the repeat testing, follow-on treatments, and artesunate dosing were also measured. With respect to the secondary set of health systems readiness indicators, they referred to the hospital availability of antimalarials, malaria diagnostics, basic equipment and job aids, and the health workers exposure to in-service trainings, guidelines and supportive supervision including levels of knowledge about standards for inpatient test-and-treat case-management.

Data were collected using Kobo Collect Toolbox mobile application and analysed using STATA version 16. Descriptive statistics formed the basis of analyses including frequencies, means and medians and inter-quartile ranges. The primary analysis, assessing the quality of inpatient malaria case-management in accordance with national guidelines, was performed at the patient level. Secondary analyses reflecting health systems readiness were undertaken at hospital and health worker levels, with the latter stratified by cadre (doctors vs nurses). Analyses were aggregated across all patient observations and stratified by hospital ownership (public vs private) and by admission wards (paediatric vs medical). The sensitivity, specificity and positive and negative predictive values of routine microscopy were calculated using standard formulas, with expert microscopists findings serving as the gold standard parasite detection. Parasite detection agreement, based on concordant results between routine and expert slide readings, was also calculated.

## Results

### Description of study populations

The study populations comprised 18 assessed hospitals, 72 interviewed inpatient health workers, 3,381 reviewed paediatric and adult admission files, and 211 rechecked routine malaria blood slides. Of the 18 hospitals, 15 were public and three were private; 16 were secondary-level and two were tertiary and teaching hospitals. Among interviewed health workers, the distribution of clinicians and nurses was nearly equal (35; 49% vs 37; 51%). The majority of interviewed doctors were male (30/35; 86%), while females were more represented among nurses (22/37; 60%). Most doctors (32/35; 91%) were medical officers. The median age of health workers was 34 years [IQR: 29–43] with a median of 6 years [IQR: 3–13] of experience in managing inpatients.

**Fig. 1:**
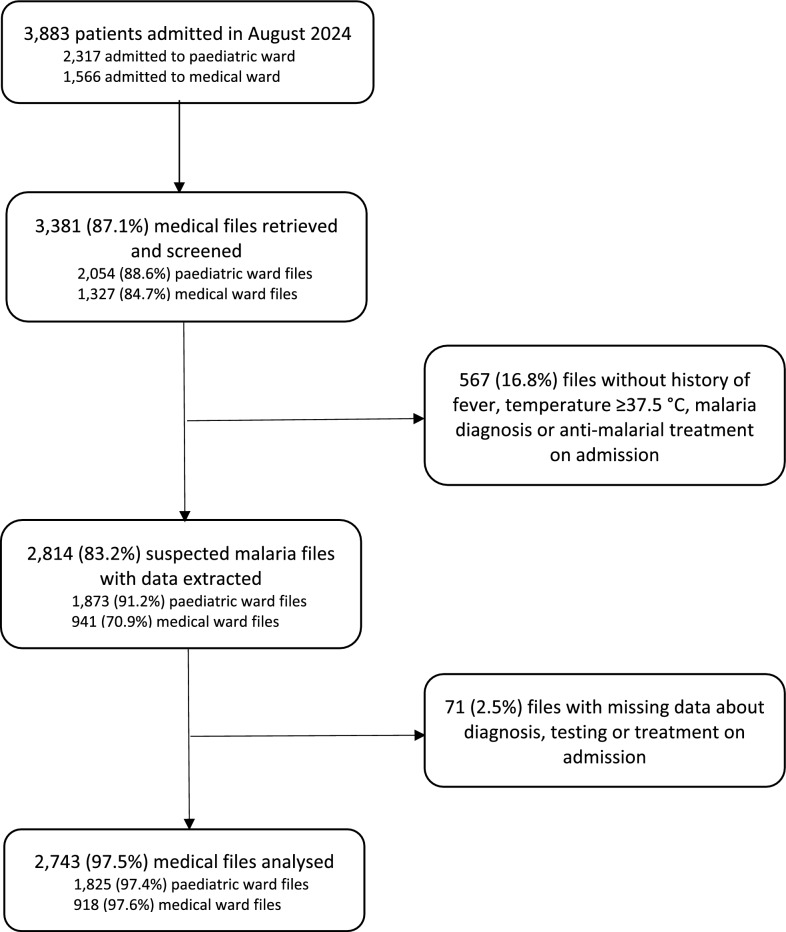
Patients’ records retrieved, screened and analysed, by admission ward

In total, 3,381 case files were screened which represented 87.1% of all admissions to paediatric and medical wards in the study period (Fig. 1). Of the 3,381 screened files, data were extracted for 2,814 (83.2%) patients who met the criteria for suspected malaria on admission. A further 71 files (2.5%) were excluded from the analysis due to incomplete data. This resulted in 2,743 suspected malaria cases analysed, with a higher proportion of paediatric than adult admissions (65.5% vs 34.5%). Private hospitals contributed 172 cases, of which nearly two-thirds were adult patients admitted to medical wards (64.0%). Female patients represented 46.7% of all inpatients and 41.5% of paediatric admissions. In private hospitals, females were more represented among adults than paediatric patients (68.8% vs 32.3%). The median age of paediatric and adult patients was 3 years [IQR: 2–6] and 40 years [IQR: 25–65], respectively. In private hospitals, the median age of children admitted to paediatric wards was 6 years [IQR: 2–9], the age greater than 3 years [IQR: 2–5] observed among children in public hospitals. The median length of admission was two days [IQR: 2–4], longer for adults than for children (3 vs 2 days) and equally two days in public and private hospitals.

Over one-third of patients with suspected malaria (36.3%) had at least one documented feature of malaria severity on admission, with similar proportions between paediatric and medical ward admissions (36.8% vs 35.3%) and slightly higher in public than in private hospitals (36.6% vs 30.8%). The most common severity features included severe anaemia (13.5%), convulsions (9.5%), prostration (7.8%), persistent vomiting (5.8%), impaired consciousness (5.3%), respiratory distress (3.3%), hypoglycaemia (2.8%), and jaundice (2.7%), while other features were documented in less than 1% of the patients. In private hospitals, the most common documented severity feature was prostration (9.9%), while no cases with documentation of severe anaemia, hypoglycaemia, or jaundice were found. Of all suspected malaria admissions, 36.5% had a diagnosis of severe malaria (documented as severe, complicated, or cerebral malaria), more commonly among paediatric (39.0%) than adult patients (31.7%) and in public compared to private hospitals (37.3% vs 24.4%). Complementing clinical severity criteria with malaria diagnosis severity documented on admission 56.0% of suspected cases met operational severity criteria.

To assess the correctness of antimalarial treatment practices for suspected malaria patients, four patient categories were constructed. The first category included severe malaria patients, defined by the presence of a positive malaria test on admission and either documentation of any severe malaria feature or a clinician-documented diagnosis of severe malaria (41.6%). The remaining three categories comprised test positive non-severe malaria patients (27.5%), test negative patients with or without severity criteria (4.7%); and patients not tested for malaria, regardless of clinical features (26.3%). The distribution of severe malaria cases was similar between public and private hospitals (41.7% vs 39.0%), but higher in children compared to adults (47.1% vs 30.6%). Children 5 years and below comprised 79.6% of severe malaria admissions in paediatric wards.

Finally, 211 routine malaria blood slides were randomly selected for rechecking at 16 hospitals. Two public hospitals did not have archived slides. Of the 211 collected slides, 137 (64.9%) were reported as positive and 74 (35.1%) as negative by routine microscopy. Of 137 routinely reported positive slides, 110 (80.3%) had a semi-quantitative “one plus” result, 21 (15.3%) had a quantitative parasite count—7 (5.1%) between 500 and 1000 p/uL of blood and 14 (10.2%) over 1000 p/uL—while 6 slides (3.4%) had no recorded parasite density, either semi-quantitative or quantitative.

### Health systems readiness

Universal availability of at least one malaria diagnostic method (RDT or microscopy) was observed, with equal availability of microscopy and RDTs at 78%, and 56% of hospitals providing both malaria diagnostic services. Injectable artesunate was in stock at all but one hospital (94%), followed by artemether (89%) and the non-recommended but widespread α-β-arteether (72%). Quinine injections were less commonly stocked (28%) while all hospitals had ACT medicines in stock, mostly artemether-lumefantrine. Triage was observed in 72% of hospitals; adult and baby weighing scales were available in 100% and 67% of hospitals respectively; however, only 28% of hospitals had a displayed artesunate administration poster (Table 1).

**Table 1:** Hospital readiness for inpatient test-and-treat malaria case-management

Hospital characteristics (N = 18)	n (%)
Availability of diagnostic services	
Any diagnostics (RDT or microscopy)	18 (100)
Non-expired RDTs in stock	14 (77.8)
Malaria microscopy	14 (77.8)
Both diagnostics (RDT and microscopy)	10 (55.6)
Availability of antimalarials	
Any injectable antimalarial in stock	17 (94.4)
Artesunate injections	17 (94.4)
Artemether injections	16 (88.9)
Quinine injections	5 (27.8)
α-β-arteether injections	13 (72.2)
Any ACT in stock	18 (100)
AL or AA in stock	18 (100)
Artemether-lumefantrine	18 (100)
Artesunate-amodiaquine	12 (66.7)
DHA-PPQ	3 (16.7)
Artesunate-pyronaridine	1 (5.6)
Availability of any malaria diagnostics, artesunate and any ACT	17 (94.4)
Availability of both malaria diagnostics, artesunate and any ACT	10 (55.6)
Availability of triage, weighing scales and treatment job aids	
Triage	13 (72.2)
Adult weighing scale	18 (100)
Baby weighing scale	12 (66.7)
Artesunate poster displayed in at least one ward	5 (27.8)

Most interviewed inpatient health workers (74%)—including both doctors (74%) and nurses (73%)—had received some form of in-service training on severe malaria case-management, either formally or through informal on-job orientation. About half (47%) had access to the latest national malaria case-management guidelines, more commonly doctors than nurses (54% vs 41%). Exposure to supportive supervision that included severe malaria management, was low overall (17%), with slightly higher rates among nurses (22%) than doctors (11%) (Table 2). Among the 12 interviewed health workers in private hospitals, all had received on-job orientation on severe malaria, but only 25% had attended formal in-service training. Regarding knowledge, the majority of health workers (72%) correctly knew that all admitted patients with fever should be tested for malaria regardless of other signs or symptoms—more commonly nurses than doctors (81% vs 63%). Less than half (43%) knew that febrile patients with a negative malaria test result should not be treated for malaria, with knowledge higher among doctors than nurses (51% vs 35%). Knowledge about artesunate treatment recommendations for severe malaria was high (82%), particularly among doctors (89%). Knowledge of ACT follow-on recommendations was lower (71%). Two-third of doctors (66%) correctly and equally identified the recommended artesunate dosing—3.0 mg/kg for children weighing less than 20 kg, and 2.4 mg/kg for patients over 20 kg. Nearly all health workers (97%) knew that three artesunate doses is a minimum dosing regimen (Table 2). Finally, health workers in private hospitals demonstrated higher levels of knowledge regarding testing criteria (83% vs 79%), treatment compliance for test negative results (67% vs 38%), and artesunate dosing (67% vs 35%).

**Table 2:** Health workers’ readiness for inpatient test-and-treat malaria case-management

	Doctors (N = 35)	Nurses (N = 37)	All HWs (N = 72)
	n (%)	n (%)	n (%)
Exposure to support interventions			
Any in-service training on severe malaria management	26 (74.3)	27 (73.0)	53 (73.6)
Formal severe malaria training	17 (48.6)	14 (37.8)	31 (43.1)
Informal on-job severe malaria training	26 (74.3)	26 (70.3)	52 (72.2)
Access to national malaria guidelines	19 (54.3)	15 (40.5)	34 (47.2)
Any supervision on severe malaria in past 3 months	4 (11.4)	8 (21.6)	12 (16.7)
Health workers knowledge [correct responses]			
Malaria testing [all fevers]	22 (62.9)	30 (81.1)	52 (72.2)
Treatment for severe malaria [AS]	31 (88.6)	28 (75.7)	59 (81.9)
Follow on treatment [AL or AA]	27 (77.1)	24 (64.9)	51 (70.8)
Treatment of test negatives [no AM treatment]	18 (51.4)	13 (35.1)	31 (43.1)
Correct AS dosing across weight groups	20 (57.1)	9 (24.3)	29 (40.3)
AS dosing knowledge < 20 kg [3 mg/kg]	23 (65.7)	17 (46.0)	40 (55.6)
AS dosing knowledge > 20 kg [2.4 mg/kg]	23 (65.7)	10 (27.0)	33 (45.8)
Minimum number of AS doses [3]	34 (97.1)	36 (97.3)	70 (97.2)

### Malaria test-and-treat case-management by admission ward and hospital ownership

Among the 2,743 suspected malaria admissions, nearly three-quarters (73.7%) of patients were tested on admissions, more commonly children than adults (79.8% vs 61.7%) and in private compared to public hospitals (84.3% vs 73.0%) (Table 3). Most of the requested malaria tests were performed (95.8%). In public hospitals, malaria microscopy and RDTs were used at similar rates (49.2% vs 51.5%). In private hospitals, microscopy was the dominant parasitological method, while RDTs were rarely used (94.5% vs 5.5%). Following malaria testing at admission, repeat testing was rarely performed (1.2%). Malaria test positivity among admitted patients was very high overall (93.7%), both in public (93.3%) and private hospitals (98.6%). Malaria microscopy demonstrated very high positivity (97.8%), while RDT positivity was somewhat lower (89.2%).

**Table 3:** Quality of inpatient test-and-treat malaria case-management, by admission ward and hospital ownership

	By admission ward		By hospital ownership		All admissions (N = 2,743)
	Paediatric ward (N = 1,825)	Medical ward (N = 918)	Public hospitals (N = 2,571)	Private hospitals (N = 172)	
	n (%)	n (%)	n (%)	n (%)	n (%)
Composite performance	834 (45.7)	243 (26.5)	1,024 (39.8)	53 (30.8)	1,077 (39.3)
Malaria test requested	1,494 (81.9)	616 (67.1)	1,965 (76.4)	145 (84.3)	2,110 (76.9)
Malaria test performed	1,456 (79.8)	566 (61.7)	1,877 (73.0)	145 (84.3)	2,022 (73.7)
Microscopy performed	757 (52.0)	304 (53.7)	924 (49.2)	137 (94.5)	1,061 (52.5)
RDT performed	707 (48.6)	267 (47.2)	966 (51.5)	8 (5.5)	984 (48.2)
Malaria test repeated	17 (1.2)	7 (1.2)	23 (1.2)	1 (0.7)	24 (1.2)
Treatment for severe malaria	N = 859	N = 281	N = 1,073	N = 67	N = 1,140
Artesunate	803 (93.5)	225 (80.1)	979 (91.2)	49 (73.1)	1,028 (90.2)
α-β-arteether	37 (4.3)	38 (13.5)	64 (6.0)	11 (16.4)	76 (6.6)
Artemether	12 (1.4)	12 (4.3)	21 (2.0)	3(4.5)	24 (2.1)
Quinine	2 (0.2)	1 (0.4)	0	3 (4.5)	3 (0.3)
ACT	3 (0.4)	4 (1.4)	7 (0.7)	0	7 (0.6)
Other AM treatments	2 (0.2)	1 (0.4)	2 (0.2)	1 (1.5)	3 (0.3)
Treatment for uncomplicated malaria	N = 527	N = 227	N = 678	N = 76	N = 754
ACT	11 (2.1)	7 (3.1)	15 (2.2)	3 (4.0)	18 (2.4)
Artesunate	449 (85.2)	123 (54.2)	528 (77.9)	44 (57.9)	572 (75.9)
α-β-arteether	34 (6.5)	67 (29.5)	82 (12.1)	19 (25.0)	101 (13.4)
Artemether	28 (5.3)	24 (10.6)	47 (6.9)	5 (6.6)	52 (6.9)
Quinine	2 (0.4)	1 (0.4)	1 (0.2)	2 (2.6)	3 (0.4)
Other AM treatments	3 (0.6)	5 (2.2)	5 (0.7)	3 (4.0)	6 (1.1)
Treatment for test negative cases	N = 70	N = 58	N = 126	N = 2	N = 128
No antimalarial	20 (28.6)	11 (19.0)	30 (23.8)	1 (50.0)	31 (24.2)
Artesunate	22 (31.4)	25 (43.1)	47 (37.3)	0	47 (36.7)
Artemether	8 (11.4)	8 (13.8)	15 (11.9)	1 (50.0)	16 (12.5)
α-β-arteether	7 (10.0)	7 (12.1)	14 (11.1)	0	14 (10.9)
ACT	12 (17.1)	7 (12.1)	19 (15.1)	0	19 (14.8)
Other AM treatments	1 (1.4)	0	1 (0.8)	0	1 (0.8)
Treatment for not tested patients	N = 369	N = 352	N = 694	N = 27	N = 721
No antimalarial	36 (9.8)	53 (15.1)	87 (12.5)	2 (7.4)	89 (12.3)
Artesunate	255 (69.1)	201 (57.1)	447 (64.4)	9 (33.3)	456 (63.3)
α-β-arteether	22 (6.0)	55 (15.6)	72 (10.4)	5 (18.5)	77 (10.7)
Artemether	29 (7.9)	25 (7.1)	48 (6.9)	6 (22.2)	54 (7.5)
Quinine	3 (0.8)	4 (1.1)	2 (0.3)	5 (18.5)	7 (1.0)
ACT	21 (5.7)	13 (3.7)	34 (4.9)	0	34 (4.7)
Other AM treatments	3 (0.8)	1 (0.3)	0	0	4 (0.6)

Regarding treatment, the use of recommended artesunate for confirmed severe malaria cases was high overall (90.2%) and even higher among paediatric patients (93.5%). However, artesunate use was lower in adults (80.1%) and particularly in private hospitals (73.1%). The most common practice discordant with treatment policy for severe malaria in private hospitals and among adult patients was the use of parenteral α-β-arteether, prescribed in 16.4% and 13.5% of cases, respectively (Table 3). ACT was rarely used for confirmed malaria patients admitted without any severe malaria criteria (2.4%). For these patients, irrational use of artesunate therapy was most common (75.9%), followed by injectable α-β-arteether (13.4%), which was increasingly prescribed for adults (29.5%) and in private hospitals (25.0%). Irrational treatment practices were also observed among test-negative patients, where a range of antimalarials—including artesunate, artemether, α-β-arteether and ACT—were often prescribed. This resulted in a test-negative compliance rate of only 24.2%, with lower compliance in adults than in children (19.0% vs 28.6%). Finally, among patients not tested for malaria, presumptive antimalarial treatment was dominant (87.7%), most commonly with injectable artesunate (63.3%), but also with other parenteral antimalarials, particularly in private hospitals (59.3%). Overall, composite test-and-treat performance—defined as a) testing of suspected malaria patients, and b) artesunate treatment for test-positive severe cases, ACT for test-positive non-severe cases, and no antimalarial for test-negative patients—was 39.3%. Performance was higher for children than adult patients (45.7% vs 26.5%) and in public compared to private hospitals (39.8% vs 30.8%) (Table 3).

### Artesunate dosing and ACT follow-on treatment practices

Table 4 shows artesunate dosing and ACT follow-on practices among all patients treated with artesunate and among those with severe malaria. Of 2,028 patients treated with parenteral artesunate who had dose specified (2,028/2,103; 96.4%), the following patterns were observed. First, nearly all patients (99.0%) were prescribed at least three doses of artesunate. Second, only 30.0% of artesunate-treated patients were prescribed ACT follow-on treatment, with the practice observed at low levels in public hospitals (26.9%) and significantly higher in private hospitals (89.2%). Third, weight was measured for only 9.1% of patients, more commonly in private than in public hospitals (22.6% vs 8.4%). While 53.7% of paediatric patients were weighed in private hospitals, this was an uncommon practice for children in public hospitals (11.0%) and for adults across both sectors (1.1%). Fourth, a 120 mg artesunate dose was prescribed for 90.3% of patients in the medical wards, with similar rates across public and private hospitals (90.9% vs 85.3%). In paediatric wards, 58.2% of children were prescribed either 30 mg or 60 mg of artesunate. Finally, similar patterns were observed among severe malaria patients, with no significant differences (Table 4).

**Table 4:** Artesunate dosing practices and follow on treatment, by ownership and admission ward

	By admission ward		By hospital ownership		All patients
	Paediatric ward	Medical ward	Public hospitals	Private hospitals	
	n (%)	n (%)	n (%)	n (%)	n (%)
All artesunate treated patients	N = 1,463	N = 565	N = 1,926	N = 102	N = 2,028
Weight measured	179 (12.2)	6 (1.1)	162 (8.4)	23 (22.6)	185 (9.1)
Artesunate 3 or more doses	1,447 (98.9)	561 (99.3)	1,907 (99.0)	101 (99.0)	2,008 (99.0)
ACT follow on treatment	336 (23.0)	273 (48.3)	518 (26.9)	91 (89.2)	609 (30.0)
Complete treatment prescribed	332 (22.7)	273 (48.3)	515 (26.7)	90 (88.2)	605 (29.8)
Artesunate treated with severe malaria	N = 778	N = 223	N = 952	N = 49	N = 1,001
Weight measured	82 (10.5)	3 (1.4)	71 (7.5)	14 (28.6)	85 (8.5)
Artesunate 3 or more doses	774 (99.5)	223 (100)	949 (99.7)	48 (98.0)	997 (99.6)
ACT follow on treatment	181 (23.3)	117 (52.5)	251 (26.4)	47 (95.9)	298 (29.8)
Complete treatment prescribed	179 (23.0)	117 (52.5)	250 (26.3)	46 (93.9)	296 (29.6)

### Accuracy of routine malaria microscopy

Of 211 rechecked blood slides, expert malaria microscopy revealed a slide positivity rate of 20.9%, substantially lower than the 64.9% positivity rate found among routine health facility slides. The sensitivity and the specificity of the routine slide results in comparison to gold standard expert microscopy were 93.2% and 42.5%, respectively. The positive predictive value (PPV) was very low at 29.9%, while the negative predictive value (NPV) was very high at 95.9%. Overall parasite detection agreement was 53.1%. The accuracy patterns between public and private hospitals were similar; however, higher rates of false positive results were observed at private compared to public hospitals (79.2% vs 68.1%) (Table 5).

**Table 5:** Accuracy of routine malaria microscopy

Routine microscopy results		Expert reading			Agreement	Sensitivity	Specificity	PPV	NPV
		Positive	Negative	Total	n (%)	n (%)	n (%)	n (%)	n (%)
All hospitals	Positive	41	96	137	112/211 (53.1)	41/44 (93.2)	71/167 (42.5)	41/137 (29.9)	71/74 (95.9)
	Negative	3	71	74					
	Total	44	167	211					
Public hospitals	Positive	36	77	113	88/168 (52.4)	36/39 (92.3)	52/129 (40.3)	36/113 (31.9)	52/55 (94.5)
	Negative	3	52	55					
	Total	39	129	168					
Private hospitals	Positive	5	19	24	24/43 (55.8)	5/5 (100)	19/38 (50.0)	5/24 (20.8)	19/19 (100)
	Negative	0	19	19					
	Total	5	38	43					

## Discussion

The quality-of-care assessment in Kano hospitals identified a series of strengths and deficiencies relevant to management of malaria in inpatient settings, across all age groups and hospital ownership types. The basic prerequisite determining hospital readiness for inpatient test-and-treat malaria case-management is the availability of recommended malaria diagnostic services and antimalarial medicines. The findings revealed that the minimum readiness standard—comprising the availability of at least one malaria diagnostic method, artesunate injections and oral ACT—was met in nearly all facilities. While such findings are intuitively expected at secondary and tertiary hospitals, they have not always been observed [19, 38] and were not seen in Kano hospitals five years earlier, when only 77% of hospitals could provide parasitological diagnosis and 59% had artesunate in stock [17]. However, despite progress in meeting the minimum readiness standards, important gaps remain in hospital capacity to deliver both diagnostic services in line with national standards, which promote prompt, bedside use of RDTs for severe malaria diagnosis and microscopy for parasite density quantifications [11]. In the absence of RDTs, hospital admission services—constrained by high patient volume, staff shortages and limited laboratory working hours—will likely provide delayed or presumptive antimalarial treatments, while the lack of microscopy precludes detection of parasitological severity criteria and treatment monitoring. Strengthening the RDT supply chain and ensuring universal access to quality-assured malaria microscopy should be prioritized in study area.

Another aspect of readiness refers to health worker support activities, such as training and supervision. The findings revealed high level of training coverage—through either formal or informal in-service modalities—but low supervisory support, and persistent knowledge gaps in key inpatient test-and-treat recommendations. Suboptimal doctors’ knowledge regarding the recommendation to test all febrile patients for malaria and comply with treatment guidance for test negative results may reflect limited access to recent updates, as well as a disregard for the standards or a perception of being superior to guidelines [39]. Future efforts to strengthen case-management should focus less on one-time educational interventions but more on continuous quality improvement strategies, with or without educational components, using context tailored packages shown to improve health worker performance [40–42].

With respect to case-management, several findings should be highlighted. First, nearly three-quarters of suspected malaria patients tested on admission and as high as 90% of severe malaria cases treated with artesunate presents major improvements compared to practices described five years earlier [17]. In 2019, compliance among the same patient categories was only 47% and 60%, respectively. Subsequently, a major increase in composite test-and-treat performance was observed. Improved availability of RDTs and artesunate in 2024 was likely a major factor contributing to these positive trends. Second, public hospitals outperformed private hospitals in the overall quality of test-and-treat case-management, primarily due to higher use of artesunate treatments for severe malaria and lower use of non-recommended treatments such as α-β-arteether. The restricted availability of free artesunate to the public sector, lower cost of alternative injectable antimalarials in the private market, non-inclusion of artesunate into all insurance schemes, weak pharmaceutical and clinical regulation, and lack of updated knowledge among prescribers regarding severe malaria treatment policies [43] may explain the inferior treatment practices found in the private hospitals. In contrast to outpatient care [44–46], the quality of inpatient malaria care in private for-profit hospitals remains largely unexamined in Africa. Future, larger quality-of-care studies, particularly in Nigeria, where health-seeking behaviour is prominent in private sector [34, 47] should prioritize this understudied area of malaria control. Third, despite the noted improvements, several case-management tasks remained poorly performed. Specifically, irrational use of antimalarials for test negative and non-severe malaria cases was widespread; repeat microscopy was absent; artesunate dosing was not based on patient weight; and ACT follow-on treatment for severe malaria was prescribed in less than a third of patients, the latter finding repeatedly reported from public hospitals in Nigeria [17, 18], across Africa [18, 20] and severely compromising patient outcomes [14]. Notably, this deficiency was not observed in private hospitals in this Kano study as similarly reported from a private not-for-profit hospital in Uganda [48]. Finally, case-management differed between age groups, with adult admissions less commonly receiving recommended test-and-treat practices. Compared to children, adults were less frequently tested for malaria, less often treated with artesunate, more commonly prescribed inferior antimalarials, and less commonly dosed based on patient weight. More positively, ACT follow-on treatment was more frequently practiced in adults, possibly reflection of longer hospital stays among adults and in-hospital ACT administration [18]. The uniform administration of 120 mg artesunate for adults inevitably result in underdosing, potentially driven by medicine availability, cost-saving considerations, ease of administration, and efforts to minimize waste. However, the irrational use of artesunate in patients who do not require it fails to address these barriers and, in addition to contributing to drug resistance, further exacerbates medicine shortages and increases treatment costs.

The accuracy levels of malaria microscopy observed in the study deserve special attention. Despite non-synchronized clinical and laboratory assessments, expert slide positivity rates were three times lower than routine microscopy and over five times lower in private hospitals—the findings raising questions about the validity of the near-universal positivity reported among study inpatients. Routine slide results showing high sensitivity and NPV, but low specificity and PPV were similar in accuracy to the findings from Kinshasa [49], yet significantly lower than performance levels reported from public facilities in Ethiopia [32, 50] and private facilities in Uganda [33]. The high rates of false positives and low rates of false negatives suggest that clinicians can be confident about routinely reported negative slides being truly negative, however less with test positive results characterized by significant overreporting. These findings are inconsistent with treatment practices, where all admitted patients with positive results, and nearly three-quarters of patients with negative results, are routinely treated for malaria. Such overdiagnosis and overtreatment practices for malaria do result in failure to treat and increased mortality among non-malaria febrile illnesses [27, 51]. Future efforts to increase the utility of malaria microscopy requires major quality investments, not only in maintaining functional equipment and adequate supplies, but also in ensuring regular on-job training, mentorship, supervision, and quality assessments [52–54]. In practice, quality-assured malaria microscopy in busy Kano hospitals should be generally reserved for severe and follow up cases, while RDTs should be used only for suspected non-severe cases—except for severe cases where RDTs can help guide prompt treatment while awaiting microscopy results. Importantly, in contrast to the current arrangement where laboratory and clinical activities operate on entirely separate platforms, laboratory personnel should be more closely linked with clinicians through joint quality improvement activities. Such activities should focus on identifying problems, planning corrective actions, continuously monitoring of practices and ultimately building of clinicians’ confidence in routine test results.

Finally, this study is not without limitations. First, purposive selection of hospitals might limit generalization of assessment results beyond the study hospitals. Second, data extractions based on medical records are often subject to documentation biases however this was unlikely for key test-and-treat data elements for which in case of absent information register cross-checks have been made. Third, the correctness of case-management was analysed from a malaria test-and-treat perspective, without accounting for management of comorbidities and malaria complications. Fourth, the quality of clinical practices was assessed in relation to national guidelines what inevitably result in misclassification of cases in absence of gold standard parasitological diagnosis. Fifth, while inpatient clinical practices were described during the peak month of malaria season, the accuracy of routine malaria microscopy was assessed during the subsequent three-month period. Finally, quality of case-management for adult patients does not include pregnant women for whom separate methods of data collection targeting maternity ward admissions are required.

## Conclusions

Despite many strengths there are still important gaps in the systems readiness and quality of inpatient test-and-treat malaria case-management in Kano hospitals in Nigeria. Non-compliance with national guidelines is often specific to the performance tasks (e.g. dose approximations, incomplete treatments, irrational use of antimalarials), patient age groups (e.g. lower testing and artesunate use for severe malaria in adults), and hospital sectors (e.g. higher use of inferior antimalarials in private hospitals). Clinicians can be confident about routinely reported negative slides but not about test positive results. Future interventions should prioritize quality assurance of malaria diagnosis, continuous clinical and laboratory quality improvement interventions and enhanced linkages between laboratory and clinical personnel by deploying joint processes of gaps identification, problem solving and practice monitoring.

## Data Availability

The datasets used and analysed during the current study are available from the corresponding author on reasonable request.
